# Silver-coated modular Megaendoprostheses in salvage revision arthroplasty after periimplant infection with extensive bone loss – a pilot study of 34 patients

**DOI:** 10.1186/s12891-017-1742-7

**Published:** 2017-09-02

**Authors:** Dirk Zajonz, Undine Birke, Mohamed Ghanem, Torsten Prietzel, Christoph Josten, Andreas Roth, Johannes K.M. Fakler

**Affiliations:** 1 Department of Orthopaedic Surgery, Trauma Surgery and Plastic Surgery, University Hospital Leipzig, Liebigstrasse 20, D-04103 Leipzig, Germany; 2 Department of Orthopaedics and Trauma Surgery, HELIOS Clinic Blankenhain, Wirthstrasse 5, D-99444 Blankenhain, Germany; 3 ZESBO – Zentrum zur Erforschung der Stuetz- und Bewegungsorgane, Semmelweisstrasse 14, D-04103 Leipzig, Germany

**Keywords:** Periimplant infection, Modular mega-endoprostheses, Silver-coated implants, Bone defects, Reinfection

## Abstract

**Background:**

Hip and knee replacements in patients with bone defects after infection correlates with high rates of reinfection. In this vulnerable patient population, the prevention of reinfection is to be considered superordinate to the functionality and defect bridging. The use of silver coating of aseptic implants as an infection prophylaxis is already proven; however, the significance of these coatings in septic reimplantation of large implants is still not sufficiently investigated.

**Methods:**

In a retrospective analysis, 34 patients who have been treated with a modular mega-endoprosthesis after a cured bone infection of the lower limb (femur or tibia) have been evaluated. One group with 14 patients (NSCG: non silver- coated group) was supplied with the non silver- coated implants: MML München- Lübeck™ modular endoprosthesis system (AQ Implants, Ahrensburg, Germany) or MUTARS® Modular Universal Tumor And Revision System (Implantcast GmbH, Buxtehude, Germany). The other group with 20 patients (SCG: silver- coated group) was supplied with the silver- coated system of MUTARS®. In addition to the clinical findings and the patients’ histories, specifically the reinfection rates, the patients’ mobility was assessed using the New Mobility Score (NMS, by Parker and Palmer).

**Results:**

The median follow-up period was 72 months, ranging from 6 to 267 months. The dropout rate was 5.8%. The reinfection rate after healed reinfection in SCG was 40% (8/20), in NSCG 57% (8/14), *p* = 0.34; α =0.05. The time for reinfection was, on average, 14 months (1–72 months) in SCG and 8 months (1–48 months) in the NSCG (*p* = 0.61; α =0.05). The two groups showed no differences in the NMS.

**Conclusion:**

With this retrospective analysis, it can be determined that the rate of reinfection of modular mega-endoprostheses on the hip and knee joint after healed periprosthetic joint infection (PJI) can be reduced by the use of silver coated implants. The time until reinfection can also be delayed by utilizing silver coated implants. Due to the low number of cases of this highly specific patient population, no statistical significance could be determined. A positive effect, however, can be assumed through the use of silver coatings in mega-endoprostheses after an infectious situation.

## Background

Hip and knee replacements are among the most common surgical procedures worldwide [1]. In response to demographic changes, the number of endoprosthetic surgeries is constantly increasing. For example, the number of total hip arthroplasties (THA) carried out in the U.S. rose by a factor of 2.5 from 200,216 in 1993 to 497,419 in 2005. In that same period, the amount of primary total knee arthroplasties (TKA) grew 1.7-fold from 135,992 to 237,645 [1–3]. With primary arthroplasty on the rise, cases of revision arthroplasty are also set to increase [4, 5]. Correlating with the number of endoprosthetic surgeries, the total amount of complications are also increasing; although, primary endoprostheses result in fewer than 10% of cases being problematic [6]. Because of this, surgeons specialising in endosprosthetics are faced with increasingly challenging situations in which conventional prosthesis systems are not sufficient, i.e. large defect situations after implant loosening with major osteolysis, periprosthetic fractures with extensive osseous substance defects, or periprosthetic infections and pseudarthrosis (non-union). A clinically established approach is the use of modular mega-endoprosthetic systems [7, 8]. The majority of experiences with this approach have been obtained through tumour surgery [9, 10]. Since modular mega-endoprosthesis systems can be modified intraoperatively, they enable solutions even in complex cases where only unsatisfactory resection arthroplasty or amputation were previously possible [4, 7, 11]. The conditions for using modular mega-endoprostheses must, however, be strictly verified. There are numerous differences when comparing primary arthroplasty with mega-endoprostheses: the implant’s larger surface area, the greater surgical access, the frequently longer duration of surgery, and the relatively higher blood loss. Also, multi-morbidity is less prevalent in patients receiving a primary arthroplasty; therefore, implantation of modular endoprostheses is associated with higher complication rates [4, 7, 12].

This is particularly the case with PJI with an implanted mega-endoprosthesis, of which a high percentage lead to ablative surgery or even death. The infection rate of modular mega-endoprostheses is stated in the literature as 4–36% [3, 13–15]. In the case of reinfections, it can rise up to 40% [4]. A possible solution is the silver coating of the endoprosthesis. Various in vitro studies have shown that silver coatings effectively inhibit or even prevent the formation of biofilms on metal surfaces of different bacteria [16, 17]. Recently, silver coating of medical devices, such as external fixation pins, heart valves, endotracheal tubes, and cardiac and urinary catheters, has been shown to reduce infection rates [18–20]. Furthermore, several clinical studies have confirmed the positive effects of silver coatings in preventing infections in endoprostheses [21–23]. This seems to be a probable method of preventing infection in particularly complex defect cases, where cured bone infections of the lower extremity are supplied with a modular mega-endoprosthesis. The aim of this study is to retrospectively evaluate patients who have been treated with a modular mega-endoprosthesis after a cured bone infection of the lower limb. The difference in the outcomes, particularly with regards to the reinfection rate, will be shown between silver- coated and non silver- coated implants.

## Methods

Prior to the start of the investigation, the local university’s ethics committee was consulted, and after examination, a positive vote was issued. The vote-number of the audit authority was 355/16-ek. Written, informed consent was obtained from all study participants, including consent for publication of the results. The diagnosis of periprosthetic infection was provided based on the international consensus for the diagnosis of periprosthetic joint infection.

Zajonz et al. [3] as reinfection or recurrence developed, a clinical and microbiological recurrence of local periprosthetic joint infections was defined, according to the antibiotic-free period and the absence of clinical symptoms for at least 6 weeks [3, 4]. Until 1999, β-lactam antibiotics (mainly ampicillin) were used as perioperative antibiotic prophylaxis. As of 1999, 2-generation cephalosporins (mainly cefuroxime) were used. Glindamycin was used in allergies.

To select the patient cohort, all patients who had been fitted with a modular endoprosthesis of the lower extremity at our hospital between September 1994 and December 2014 were retrospectively identified. Patients’ data was collected based on their archived records and electronic files in IS-H SAP (Siemens AG Healthcare Sector, Erlangen, Germany), as well as radiological findings and images from SIENET MagicWeb/ACOM (Siemens AG Healthcare Sector, Erlangen, Germany). All available data was acquired from our patient documentation system. All patients were invited to and consulted for a clinical follow-up. Patients who did not appear for a follow-up were interviewed by telephone. The patients who could not be examined either in person or by telephone made contact with their family doctors. In addition to the clinical findings and the patient’s history, the NMS was used as an assessment of mobility [24].

**Fig. 1:**
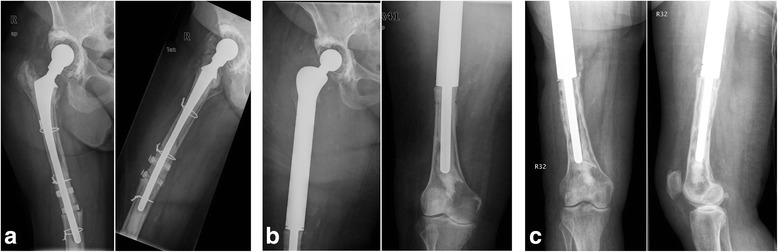
Anterior-posterior and lateral X-rays of the right hip with thigh of a 78-year-old woman with **a** a total hip arthroplasty (THA) after multiple revisions and acute periprosthetic fracture with implant loosening **b** after implantation of a proximal femur replacement and intraoperative detection of staphylococci with the clinical presentation of a PJI with implant-preserved revision and antibiosis **c** after 14 months, a chronic infection with increasing loosening of the implants

**Fig. 2:**
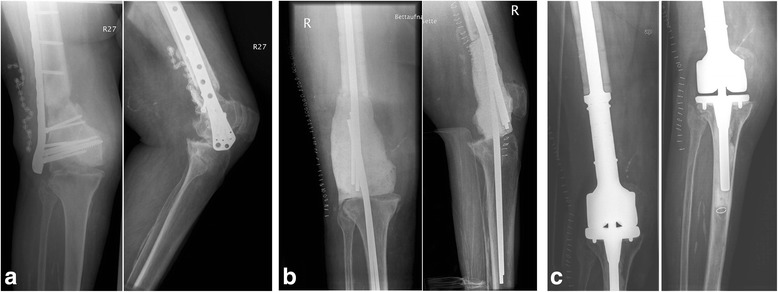
Anterior-posterior and lateral X-rays of the right knee with thigh of a 74-year-old woman with **a** infected pseudoarthrosis of the right distal femur after plate osteosynthesis of a supracondylar femur fracture **b** resection of the distal femur and implantation of an intramedullary cement spacer **c** after implantation of a silver- coated distal femoral replacement endoprosthesis (System MUTARS® Modular Universal Tumor And Revision System, Implantcast GmbH, Buxtehude, Germany)

From the patient population, a total of 36 patients with implanted modular endoprostheses after cured infections were identified. Due to inadequate documentation, 2 patients were excluded. From these 34 patients, two groups were formed. One group (NSCG: non silver- coated group) with 14 patients, 8 of whom were supplied with the non- silver- coated system: MML München- Lübeck™ modular endoprosthesis system (AQ Implants, Ahrensburg, Germany) and 6 with non- silver- coated system MUTARS® Modular Universal Tumor And Revision System (Implantcast GmbH, Buxtehude, Germany) [4]. Figure 1 of those, 3 were knee and 11 were hip endoprostheses. The other group (SCG: silver- coated group) consisted of 20 patients, all of whom were supplied with the silver- coated system MUTARS® Modular Universal Tumor And Revision System (Implantcast GmbH, Buxtehude, Germany). This group consisted of 7 knee and 13 hip endoprostheses. Figure 2 All patients had an infection of the lower extremity (prosthesis infection, infection osteosynthesis, and secondary infections after a periprosthetic fracture), which was treated with either spacer implantation or removal of the material (Girdlestone arthroplasty). The handlingof the infection was carried out for all patients on the basis of the standards for treatment of periprosthetic joint infections according to the current guidelines of our hospital. In all patients, the infected implants were removed and reimplantation took place after infectious conditioning. An antibiotic treatment was administered under microbiotic and pharmaceutical stewardship.

Statistical analysis was performed using the spreadsheet software Microsoft Excel (Microsoft Corporation, Redmond, USA). Both examination groups are normally distributed and not connected. Both groups were normally distributed, as determined by optical analysis of the distribution pattern. For the same variance, the *t*-test for two unconnected samples was used to calculate significance. The chi-square test according to Pearson was used for nonlinear test variables, with the level of significance being set at 5% (α = 0.05). Statistical advice from a mathematician has been sought.

## Results

The median follow-up period was 72 months, ranging from 6 to 267 months (SCG: 72 months (6–114) and NSCG: 74 months (18–267)). In two patients, no further information could be obtained despite extensive research (telephone consultation, written inquiry, consultation with the family physician). Thus, the drop out rate was 5.8%. Due to the lengthy follow-up period, 13 patients had already passed away prior to the latest follow-up examination. Extensive examination of the remaining patients (consultation with family members and doctors, as well as file analysis) proved there was no reinfection to be found; all patients had died of other causes (pulmonary embolism, tumor progression, cardiac genesis, age-related multimorbidity). In these patients, the last file entry in our hospital was defined as the follow-up time.

The patient specifics of the two groups, such as age, gender, and secondary diseases, are presented in Table 1. There was no significant difference between the groups in any of the features. Additionally, there was no fundamental difference in the treatment regime between the two groups. The individual treatment strategy (antibiosis, number of revisions, Spacer vs. Girdelestone) had been adapted to accommodate the specific situation of each patient. The specifics for the infection repair before reimplantation are shown in Table 2. There was no significant difference between the groups in any of the features either. The observed bacteria in the groups are shown in Table 3. The reinfection rate after healed reinfection in SCG was 40% (8/20). In the NSCG, it was 57% (8/14). A statistically significant difference was not detectable (*p* = 0.97; α =0.05). The time for reinfection was, on average, 14 months (1–72 months) in SCG and 8 months (1–48 months) in the NSCG (*p* = 0.61; α =0.05). In both groups, 37% (3/8) showed a germinal change to initial infection. The two groups showed no differences in the NMS, used as an assessment for mobility. The exact distribution is shown in Table 4.

**Table 1:** Patient specifics of the groups, such as sex, median age (min-max), diabetes, immunosuppression due to medication, peripheral arterial disease, malignant tumour, anticoagulation, and rheumatism, are presented

	Silver- coated group SCG)	non silver-coated Group (NSCG)	Statistical significance (α = 0.05)
Femal sex	55% (11/20)	57% (8/14)	0.91
Median Age (min-max)	74 (46–83)	69 (35–87)	0.36
Diabetes	45% (9/20)	42% (6/14)	0.91
Drug immunosuppression	5% (1/20)	7%(1/14)	0.80
Peripheral arterial disease, PAD	10% (2/20)	0	0.21
Malignomas	0	14% (2/14)	0.08
Anticoagulation	30%(6/20)	28%(4/14)	0.93
Rheumatism	5%(1/20)	7%(1/14)	0.85

**Table 2:** Specifics for the infection repair before reimplantation, such as multiple-stage prosthesis replacement with bone cement spacer or temporary girdlestone, as well as one-stage prosthesis replacement, number of revisions up to reimplantation (median; min -max), time until reimplantation after initiation of infection therapy in months, reimplantation time in minutes, time spent in hospital for treatment, C- reactive protein (CRP) at discharge (mg/dl), Leukocytes at discharge (10exp9)

*Median (min -max)*	Silver- coated group (SCG)	non silver-coated Group (NSCG)	Statistical significance (α = 0.05)
Multiple-stage prosthesis replacement (bone cement spacer)	16/20 (80%)	10/14 (71%)	0.81
Multiple-stage prosthesis replacement (temporary girdlestone)	2/20 (10%)^a^	1/14 (7%)	0.71
One-stage prosthesis replacement	4/20 (20%)	3/14 (21%)	0.61
Number of revisions up to reimplantation (median; min -max)	4 (0–10)	4 (0–7)	0.19
Time to reimplantation after initiation of infection therapy in months	3 (0–10)	4 (0–24)	0.28
Reimplantation time in minutes	188 (128–236)	193 (122–277)	0.51
Time spent in the hospital for infectious treatment	22 (10–139)	32 (14–158)	0.74
C- reaktive proteine (CRP) at discharge (mg/dl)	17.5 (4.3–89)	18.2 (1.5–89)	0.39
Leukocytes at discharge (10exp9)	7 (5–10.5)	6,6 (4.7–10)	0.92

^a^In two cases, both temporary Girdlestone situations and cement spacer therapy were used

**Table 3:** Observed bacteria in the groups (MRSA: methicillin resistant *Staphylococcus aureus*)

Bacteria	Silver- coated group (SCG)	non silver-coated Group (NSCG)
*Staphylococcus aureus*	8 /20 (40%)	2/14 (14%)
of this MRSA	5/20 (25%)	0
Coagulase negative staphylococci	7/20 (35%)	4/14 (28%)
of this *Staphylococcus epidermidis*	2/20 (10%)	4/14 (21%)
Enterococci	6/20 (30%)	4/14 (21%)
Pseudomonas	3/20 (15%)	1/14 (7%)
*Escherichia coli*	4/20 (20%)	1/14 (7%)
without germination	4/20 (20%)	5/14 (35%)
mixed infections	12/20 (60%)	4/14 (28%)

**Table 4:** New Mobility Score (Parker and Palmer) as assessment for mobility of the two groups (SCG vs. NSCG) and statistical significance

Mobility	Silver- coated group (SCG)	Non silver-coated Group (NSCG)	Statistical significance (α = 0.05)
Able to get about the houase	2 (1–2)	2 (0–3)	0.95
Able to get aout of the house	2 (0–2)	2 (0–2)	0.42
Able to go shopping	1 (0–2)	1,5 (0–2)	0.41
Total score	5 (1–6)	5,5 (0–7)	0.50

In each subgroup, a maximum of 3 points (3 no difficulties, 2 with an aid, 1 with help from another person, and 0 not at all) can be achieved. A maximum score of 9 points can be achieved

## Discussion

Periprostetic joint infections of mega-endoprostheses remain a serious complication in orthopedic surgery [4]. Furthermore, reinfection is an escalating problem with revision surgery in patients who suffered from infections associated with primary endoprosthetic replacement of the knee and hip joint. These patients may need multiple revision surgeries and in some cases, even amputation [4, 7, 9]. Many different approaches to treatment have been pursued. Nevertheless, the reinfection rate is still around 40% [4]. One approach to therapy is the use of silver coated implants. Since ancient times, people have been aware of the antimicrobial effect of various metals (i.e. silver and copper), but the exact methodology has yet to be investigated. The reason for the antimicrobial potency of the different ions appears to be the oligodynamic effect [25]. First, clinical trials from an orthopedic background were carried out through animal experiments. Gosheger et al. investigated the infection rates and the side effects of silver- coated implants (MUTARS) versus titanium implants in rabbits. The silver group showed significantly lower infection rates (7% versus 47%, *p* < 0.05) in comparison with the titanium group [26]. Usually, the non-scientific press emphasizes the toxic effect caused by the release of silver ions from silver-coated implants; however, the concentrations of silver in the blood in both the animal and human trials did not reach toxic levels. Even in histopathological investigations of the tissues, histological changes of the organs could not be determined. In conclusion, silver-coated mega-endoprostheses allow a release of silver, without any presentation of local or systemic side-effects [26, 27]. In clinical research, Hardes et al. showed a substantial reduction of periprosthetic infection of mega-endoprostheses in patients with bone sarcoma from 17.6% (51 patients) in the titanium group to 5.9% (74 patients) in the silver group over a 5-year period [20]. Also, a recent study by Grosso et al. showed a significant reduction in PJI with the use of silver-impregnated occlusive dressing in over 1100 primary endoprostheses on the hip and knee (1,58% to 0,33%, *p* = 0.03). These studies, however, deal with previously uninfected areas. Despite extensive literature research, work dealing with the PJI rate after pre-existing infections in endoprostheses, especially mega-endoprostheses, could not be found. Only Wafa and colleagues reported their experiences in a case-control study of the incidence of early periprosthetic infection with silver-treated endoprostheses in high-risk patients (primary reconstructions, one-stage revisions and two-stage revisions for infection) [28]. In this study, the overall post-operative infection rate of the silver-coated group was 11.8%, in contrast to the 22.4% for the control group (*p* = 0.033). Wafa concluded that silver-treated implants were particularly useful in two-stage revisions for infection and in those patients with incidental positive cultures at the time of the prosthetic’s implantation. Debridement with antibiotic treatment and retention of the implant appeared to be more successful with silver-coated implants [28]. Especially in cases of treated PJI, the reinfection rate with non-silver coated endoprostheses was 20%, whereas, it reached up to 40% with mega-endoprostheses. Based on the available analysis and the successful results in aseptic endoprostetics, a positive effect in PJI can also be assumed. Similarly in our analysis, there was a reduction of PJI from 57% to 40% through the use of silver coatings. However, these results were not statistically significant, due largely to the low number of cases of this highly specific patient population. Additionally, the time until reinfection was almost twice as long in the SCG compared to the NSCG (14 vs. 8 months). It is striking that in our study, mostly *Staphylococcus aureus*, specifically 25% of which was MRSA, was found as the initial germ in the SCG. Table 2 It is known that PJI with MRSA due to an aggressive biofilm has a significantly higher failure rate than other non-resistant germs. Thus, the lower SCG reinfection rate can be given more importance in this difficult germination. It can therefore be assumed that the formation of a biofilm in silver coating will be at least reduced, if not prevented. Hazer et al. could also show this effect in the rabbit model [29]. A significant difference in the germ spectrum was not established between the two groups. Exact statements regarding the germination parameters are only possible to a limited extent due to the small case number. It is noticeable that in both groups, a change of the germination had occurred in one third of the patients. This indicates the presence of mixed infections or superinfections, which is not unusual for this vulnerable patient population [4]. The coating had no effect on the germination specimen in our investigation. There are no valid statements in prior research regarding this issue.

### Limitations

A disadvantage of this study is the low number of cases, which is a highly specific and rare patient population. Therefore, only tendencies, rather than statistically valid statements, are possible. The inhomogeneity of the treatment is also seen as a limitation, whereby the treatment of PJI is always case-dependent; nevertheless, this can be put into perspective by the comparable treatment modalities (Tables 1 and 2) and the same standards in treatment. Due to the long study period of 6 years (median 72 months) and the advanced age of many of the patients, a significant number had already died. Due to the retrospective survey occurring over a long period of time, details of the survey also got lost (antibiotics, sampling, etc).

## Conclusion

Through this retrospective analysis, it can be determined that the rate of reinfection of modular mega-endoprostheses on the hip and knee joint after healed PJI can be reduced through the use of silver coated implants. The time until reinfection can also be prolonged using silver coated implants. Due to the low number of cases of this highly specific patient population, no statistical significance could be determined; however, a positive effect can be assumed from the use of silver coatings in mega-endoprostheses after an infection develops.

## Abbreviations

MRSA: Methicillin resistant *Staphylococcus aureus*
NMS: New Mobility Score
NSCG: Non silver- coated group
PJI: Periprosthetic joint infection
SCG: Silver- coated group
THA: Total hip arthroplasty
TKA: Total knee arthroplasty
